# 5-year safety and efficacy of the once-daily antiretroviral regimen of efavirenz (EFV)/emtricitabine (FTC)/tenofovir disoproxil fumarate (TDF)

**DOI:** 10.1186/1758-2652-13-S4-P6

**Published:** 2010-11-08

**Authors:** A Lazzarin, M Johnson, E Ribera, L Weitner, SS Chen, DR Warren

**Affiliations:** 1 Hospital St Raffaele Scientific Institute, Milan, Italy; 2 Royal Free Hospital, London, UK; 3 Hospital Vall D'Hebron, Barcelona, Spain; 4 IPM Study Centre, Hamburg, Germany; 5 Gilead Sciences, Inc, Foster City, California, USA

## Background

The goal of highly active antiretroviral therapy (HAART) is to suppress HIV RNA to undetectable levels over many years and is primarily dependent on adherence, which is aided by using a once daily regimen with good tolerability and low pill burden. In Study 934 the time to discontinuation for the twice daily regimen of EFV qd + zidovudine/lamivudine bid was significantly shorter than for the once daily regimen (EFV+FTC+TDF) (p=0.003). Herein are the 5 year safety and efficacy data for this once daily regimen.

## Methods

160 subjects (89% male, 64% white, mean age 41 yrs) in Study 934 originally randomized to the once daily regimen of EFV+FTC+TDF who completed 144 weeks agreed to switch to the single tablet formulation (EFV/FTC/TDF) and remain on study for an additional 96 weeks for a total of 240 weeks.

## Results

At baseline (BL), mean HIV RNA= 5.03 log10 c/mL, mean CD4 count= 243 cells/mm3, and 88% had symptomatic HIV or AIDS. After 240 weeks of follow-up:

87% had HIV RNA <400 c/mL and 84% <50 c/mL (M=F); mean CD4 cell increase from BL= 346 cells/mm3. The mean (range) adherence rate was 97% (83-100%). Seventeen subjects discontinued EFV/FTC/TDF: withdrew consent (6); lost to follow-up (5); adverse events (2: osteoporosis (1) and anal cancer (1)); incarceration (2); non-adherence (1); and relocated (1). No patient discontinued due to renal adverse events. Mean change from BL in estimated glomerular filtration rate (e-GFR) by Cockcroft-Gault was -7 mL/min (Mean BL e-GFR, 129 mL/min).

## Conclusions

Through 240 weeks, the once daily HAART regimen of EFV+FTC+TDF (dosed as single tablet regimen, EFV/FTC/TDF, from Week 144-240) demonstrated durable antiretroviral efficacy and immunologic recovery in antiretroviral-naïve patients. The decline in e-GFR was mild and not clinically significant.

